# Muscular imbalances and balance capability in dance

**DOI:** 10.1186/s12995-018-0218-5

**Published:** 2018-12-03

**Authors:** Eileen M. Wanke, Julia Schreiter, David A. Groneberg, Burkhard Weisser

**Affiliations:** 1 Institute of Occupational, Social and Environmental Medicine, Goethe-University Frankfurt Main, Theodor-Stern-Kai 7, 60590 Frankfurt am Main, Germany; 2 Department Sports Medicine and Training Science, Christian-Albrechts-Universität Kiel, Institute of Sports Science, Olshausenstrasse 74, 24098 Kiel, Germany

**Keywords:** Formation dance, Laterality, Motor skills, Injuries

## Abstract

**Objective:**

A high unilateral load to the musculoskeletal system is specific for formation dance. Due to the lack of data the aim of this study was the side-related (right – left) analysis of strength- and balance capability subject to injuries, gender and performance standards.

**Methods:**

*N* = 51 dancers (m: *n* = 24, f: m = 27) of two performance levels participated in this cross-sectional study. Double-sided tests of the isometric maximal strength of relevant muscle groups and the balance capability were carried out. The tests were supplemented by a self report questionnaire.

**Results:**

Tests of the isometric maximal strength in the elite performance level showed significant differences between either side of the body. As to the balance capability, no significant side-related differences could be found in. Correlations between the strength capability and the injuries could be observed in either group.

**Conclusion:**

The significant strength differences are presumably caused by the right-sided load in the dance-specific movements. The cautious conclusion that movement patterns challenge the stability of either side of the body likewise may be allowed. The increased injury frequency at the muscularly stronger side of the body primarily results from an overload. An additive muscular training should be considered as a preventive measure.

## Background

Dance sport is the competition- and performance-oriented variant of ballroom dancing. Within dance sport, which is one of the technical/compositional and aesthetic sports, formation dance is a further discipline in addition to single-pair dance [1]. As a result of the 1950s, formation dance sport has become established in its present form with a 5-level league system and annual national and international championships. As in single pair dancing, a distinction is made between standard and Latin American formations. The aim of formation dancing is to have the 8 couples of a team moving synchronously and precisely on the dance floor during the five Latin American dances (Samba, Rumba, Jive, Cha-Cha-Cha, Paso Doble) [2].

**Fig. 1:**
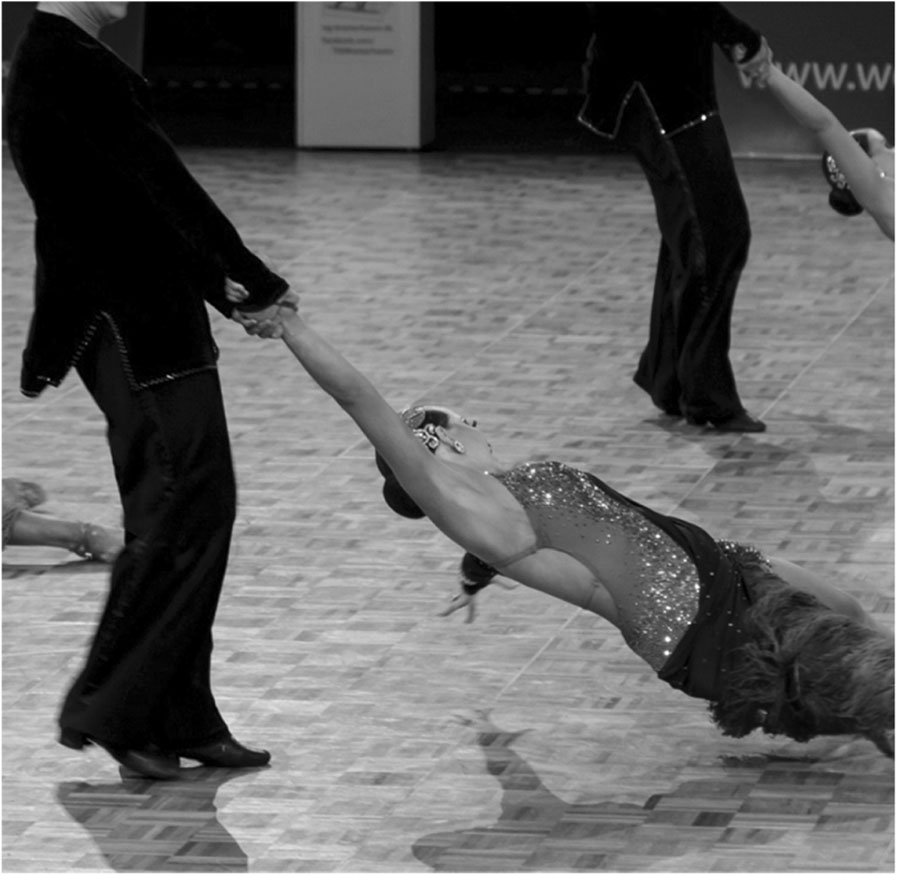
“slingshot” as a characteristic element of movement in Latin American formation dance with one side loaded (Photo: Oldenbüttel)

In contrast to the single pair dance, a performance in formation dance lasts six minutes, 4.5 min of which are allotted to the main part relevant for the evaluation with the remaining 1.5 min to the marching in and out with. The cardiovascular stress extends in the fast Latin American dances (e. g. Jive) into the maximum stress range, with high demands being conductive to injuries [3, 4]. In contrast to single pair dance, there are formation dance-characteristic elements of movement in Latin American formation dance that go beyond the dance steps, the gender-specific tasks and movement and posture characteristics which are either more frequent (e. g. the *Lankenau* pirouette, *Cha-Cha-spins*) or they are found exclusively in the Latin American formation dance (e. g. *round-about, potstirrer*) (Fig. 1). These steps are often key elements in the rating during a competition for they require a high strength capability and major coordination skills [5]. In addition to their high movement dynamics, these characteristic elements are associated with a one-sided right-sided load on the movement and support system of either dance partner and characteristically initiated and terminated by the male partner in each case as well as predominantly presented unilaterally (on the right) [6, 7]. In combination with the already open dance posture performed on the right, this results in a laterality of movements in favor of the right side.

In the last few years the formation dance has changed a lot [7]. Speed and dynamics have increased [8]. Numerous flowing position changes without a complete standstill as well as the trend towards a direct line-up of several extreme difficulties have further increased the demands on dance technique, the musculoskeletal as well as the cardiovascular system. This development seems to be necessary in order to remain competitive both on nationally and internationally. In addition to the high, often one-sided, loads, this results in a training time of up to 15 h weekly combined with only occasional professionalism and a not to be underestimated subordination of individual physical prerequisites to the benefit of group performance [9].

### Objective of the present study

There are characteristic stylistic elements in formation dance associated with unilateral loads on the body. While there are studies on other dance directions indicating a morphological laterality according to a higher right-sided force level [10–12]. As a consequence of the gender-specific and dance direction-specific movement contents, there have been no studies on dance sport in this respect. Hitherto, it is largely unclear to what extent these asymmetric loads lead to morphological and/or functional adjustments [13]. At that, a correlation with the frequency of injuries on a certain side of the body has not yet been sufficiently investigated. Studies on the side-related balance capability of the body, as well as the relation to develop the strength capability of either side of the body have also been lacking. However, it cannot be ruled out that the named factors favor the occurrence of traumatic (acute) injuries as well as the inappropriate loads and overloads of the musculoskeletal system [14]. Mergenthaler [15] and Strauss [16, 17] have already ranked formation dancing as the discipline with the highest injury risk within the various dance sports disciplines. Wanke et al. [9] observed a possible correlation between gender-specific movement elements and injuries or chronic sports injuries in formation dance and pointed to a further need for research in this discipline.

The aim of the present study is therefore to analyze the balance as well as the maximal isometric strength capability (hamstrings, quadriceps, triceps, biceps, shoulder elevators) in formation dancers regarding body sides, injury frequency, gender and performance standard (performance class: EPL; junior level: JL).

## Methods

### Subjects

Table 1 shows the descriptive data of the participants. The study included a total of *n* = 51 formation dancers with an average age of 21.6 (± 4.0) years and an average height of 174.5 (±10.3) cm. The mean body mass index (BMI) and body weight (kg) were 21.8 (±2.1) and 66.6 (±11.1) kg, respectively. Only significant differences were found between the male and female dancers but not between the JL and EPL level groups. The junior level dancers (JL, regional league or lower) consisted of *n* = 29 dancers with the performance level dancers (EPL, highest national league) consisting of *n* = 22 dancers. All participants were healthy at the time of the examination and took part in competitions as members of a dance formation.

**Table 1:** Descriptive data of the group of test subjects

Test subjects (n)	Age in years M(SD)	Height in cm M(SD)	Weight in kg M(SD)	Body Mass Index (BMI) M(SD)
All (*n* = 24) Male	22.8 (±5.0)	182.8 (±7.6)	74.6 (±9.7)	22.3 (±2.0)
All (*n* = 27) Female	20.5 (±2.4)	167.2 (±5.6)	59.5 (±6.5)	21.3 (±2.0)
EPL all (*n* = 22)	21.7 (±2.2)	175.4 (±11.5)	67.4 (±12.4)	21.8 (±2.0)
EPL (*n* = 11) Male	22.2 (±2.2)	184.5 (±8.4)	75.2 (±11.5)	22.0 (±2.1)
EPL (*n* = 11) Female	21.3 (±2.2)	166.2 (±4.6)	59.7 (±7.5)	21.6 (±2.0)
JL all (*n* = 29)	21.5 (±5.0)	173.9 (±9.4)	65.9 (±10.2)	21.7 (±2.1)
JL (*n* = 13) Male	23.4 (±6.6)	181.4 (±6.8)	74.1 (±8.3)	22.5 (±2.0)
JL (*n* = 16) Female	19.9 (±2.4)	167.8 (±6.3)	59.4 (±6.0)	21.1 (±2.1)

Mean value (M) and standard deviation (SD)

### Balance measurement

The MFT S3-Check device (measurement of stability and sensorimotor index, symmetry ratio) was used to measure the balance capability and body stability. This measuring device consists of an unstable disc, equipped with sensors that detect all movements of the subjects. As soon as the centre of gravity was removed from the center of the disc, the measuring plate tilted to a maximum of 12° towards the pre-determined direction. The test subject’s task was to re-stabilize the standing base as quickly as possible and to keep it as immobilized as possible for a period of 30 s. Deviations from the so-called optimum state (parallel to the ground) were registered by means of acceleration and inclination sensors. The measuring plate was connected to computer software via an USB interface and could be evaluated. The better the sensorimotor regulation capability, the better the sensorimotor index was assessed. The balance of the movement deviations to the different directions was given with the symmetry ratio. The stability index was composed of the two values described above and evaluated the body stability while standing on an unstable base. Within the scope of this study, the following measurements were carried out (2 in each case with the better one evaluated):

- Right - left - right (R-L, sagittal plane, double-legged)
- Forward and backward (F-B, frontal plane, double-legged).

For bipedal measurements, validated reference values were available [18].

### Measurement of the isometric maximum force

The measurements were carried out with the device “*m3 Diagnosis professional*” whose use corresponds to scientific quality criteria [19, 20] from *SCHNELL Trainingsgeräte GmbH*. After correct individualized adjustment of the device, the test subject was instructed to reach the individual maximum force within a period of 5 s. Alternately, the right and left sides were tested three times and the best value was recorded. The following relevant muscles or muscle groups were examined:

- Upper extremity and shoulder: M. biceps, M. triceps, shoulder elevators
- Lower extremity and pelvis: *M. quadriceps*, ischio-crural muscles (hamstrings)

Five muscle groups were chosen for the specific movements in formation dance in order not to overstrain the subjects. The selection was made according to relevance and feasibility to ensure a comparison of the results.

### Questionnaire

The questionnaire was used to collect anthropometric data, as well as acute injuries and chronic complaints (including behavior, localization, type, presumed cause, therapy or form of treatment, respectively) according to de Marees [21] by comparing both body sides over a period of 24 months as recommended by Liederbach et al. [22]^.^ and modified according to Wanke et al. [23]. These data were correlated with the force values and the results of the muscle function tests. All data have been collected in strictly pseudonymized form.

### Data analysis

The evaluation was carried out with SPSS (IBM SPSS Statistics 21). Frequency distributions were recorded on the basis of descriptive statistics and graphically displayed. Difference hypotheses were verified by t-tests for dependent or independent samples (right-left). The Levene test was used to check the mean values for variance homogeneity. The calculation for correlations of force differences in lateral comparison and injury patterns was carried out as a bivariate correlation according to Pearson. The significance level was determined at *p* < 0.05. For the simultaneous observation of a laterality of the parameter, the respective difference (right - left) of the variable was calculated using the data with the corresponding sign (− = left-sided).

In the calculation of the correlations between strength capability and injuries, injuries with different force variables (type of the examined musculature as difference between the right and left side of the body) were carried out.

A positive ethics vote of the ethical committee of the university had been obtained before the studies began. All volunteers gave informed consent to the tests.

## Results

Table 2 shows the differences in the isometric maximum force between the right and left side of the examined musculature. Significant force differences were found almost exclusively in the area of the upper extremity (UE) as well as in the dancers of the EPL (all examined muscle groups of the UE) and the JL (2 out of 3 examined muscle groups of the UE). For the male dancers, only the differences in the area of the M. triceps were significant. There were hardly any significant differences in the lower extremity. With one exception, a dominance of the right side of the body could be observed.

**Table 2:** Differences in maximum isometric strength between the right and left side of the body (*n* = 51)

Muscle (group)		Test group	Number (n)	*t*-value	*p*-value	Dominating side
Upper extremity	M. biceps	EPL male	11	−1.22	0.256	
		EPL female	11	−7.4	< 0.001**	right
		JL male	13	−0.32	0.753	
		JL female	16	−5.84	< 0.001**	right
	M. triceps	EPL male	11	−2.95	0.014*	right
		EPL female	11	−3.82	0.003*	right
		JL male	13	−1.2	0.255	
		JL female	16	−1.23	0.239	
	Shoulder elevators	EPL male	11	−0.20	0.844	
		EPL female	11	2.471	0.033*	right
		JL male	13	1.38	0.193	
		JL female	16	2.83	0.013*	right
Lower extremity	*M. quadriceps*	EPL male	11	1.93	0.083	
		EPL female	11	1.10	0.297	
		JL male	13	−2.52	0.027*	left
		JL female	16	1.28	0.219	
	Ischiocrural muscle group	EPL male	11	1.93	0.082	
		EPL female	11	−1.45	0.177	
		JL male	13	0.74	0.473	
		JL female	16	0.90	0.403	

* *p* < 0.05, **P < 0.001

### Balance capability

Although the female dancers in both groups (achieved a better value in the bipedal balance measurement than the male test subjects of the respective grouping, the differences in the EPL group were not significant (All: R-L: *p* < 0.001, F-B: *p* = 0.024; EPL: R-L: *p* = 0.14, F-B: *p* = 0.76; NL: R-L: *p* = 0.002, F-B: *p* < 0.001).

### Injuries

A total of 114 injuries could be identified in 51 subjects on the basis of the questionnaire. Chronic injuries (44.7%) were less frequent than acute injuries (55.3%). The diseases and injuries were relatively evenly distributed between EPL (*n* = 61) and JL (*n* = 53), but all female dancers reported more injuries than their male counterparts. Chronic injuries were predominant in the EPL with acute injuries (Table 3) in the JL.

**Table 3:** Differences in injury frequency between the right and left side of the body

	Type of injury			Side differences		
Test group	Acute (n)	Chronic (n)	Total (n)	Acute (side)	Chronic (side)	Total (side)
EPL male	14	12	26	0.031* (r)	0.026* (r)	0.015* (r)
EPL female	14	21	35	0.553	0.211	0.255
JL male	11	4	15	0.436	0.082	0.753
JL female	24	14	38	0.270	0.669	0.453
Total	63	51	114			

* *p* < 0.05, right: r, left: l

Within the JL, no significant differences regarding the injury frequency (chronic and acute as a whole) could be found in comparing the right and left side of the body. In the EPL, on the other hand, differences were found between the two sexes compared to the JL (acute: *p* = 0.038), chronic: *p* = 0.015). From a gender-specific point of view, however, these differences were significant only in the male dancers.

As a whole, the lower extremity was most frequently affected with 20.2% of ankle joint injuries. This was followed by hand and wrist injuries with 19.2% and by foot and shoulder injuries with 11.7% each. The localizations significantly differed between female and male dancers particularly at the right side of the hand (m > f, *p* = 0.013), the shoulder (m > f, *p* = 0.017) and hip (f > m, *p* = 0.032) being higher on the right side (m vs. f, *p* = 0.009). Taking into account gender and level of performance, the right shoulder of the EPL male dancers was injured more often (*p* < 0.025) than the right shoulder of the JL male dancers. The same was observed with the right hand (EPL male and female) in relation to the JL (*p* = 0.021). All further calculations did not result in any significant differences with respect to a preferred side for certain injury localizations. Therefore, no significant gender- or performance-related differences within the JL dancers were found.

### Correlation between balance capability and injury frequency subject to the body side

In the EPL, no correlations between injuries and balance could be calculated. In the JL, it turned out that a poorer capability to balance was associated with the development of chronic overload damage on the right side (*p* = 0.028; *r* = 0.605). The same was valid for the female dancers of the JL (*p* = 0.045 and *r* = 0.507).

### Correlation between strength capability and injury frequency subject to the body side

Numerous significant correlations were observed with the injuries.

EPL males: There were clear, mostly negative correlations. Thus, the greater right-sided maximum strength of the hamstrings of the EPL male dancers correlated with an increased injury frequency on the contra lateral, weaker side (with injuries overall: *p* = 0.028, *r* = − 0.656; acute injuries: *p* = 0.025, *r* = − 0.667; injuries to the lower extremities: *p* < 0.001, *r* = − 0.878). Furthermore, the same group showed a significant correlation between the stronger right side muscle strength in the M. biceps region and the number of injuries on the left, weaker, side of the body (*p* = 0.027, r = − 0.66).

EPL females: Here, too, there was a clear correlation between the stronger (mostly right-sided) muscle strength on the one hand and the occurrence of injuries on the weaker, contra lateral left side of the body as negative correlations on the other (Table 4).

**Table 4:** Correlations between balance, isometric maximum strength of selected muscle groups and injuries/chronic damages/complaints of the EPL (*n* = 22)

		Vtotal		Vacute		Vchron		Vuex		Vtor	Vlex	
		m p (r)	f p (r)	m p (r)	f p (r)	m p (r)	f p (r)	m p (r)	f p (r)	m p (r)	m p (r)	f p (r)
Balance	Stability r – l	0.810	0.419	0.497	0.837	0.682	0.133	0.183	0.177	0.845	0.561	0.986
	Stability forward – backward	0.884	0.847	0.515	0.790	0.571	0.592	0.263	0.595	0.781	0.264	0.813
	Stability total	0.945	0.440	0.959	0.692	0.943	0.101	0.111	0.129	0.968	0.288	0.839
Strength	M. biceps	0.066	0.397	.084	0.801	0.186	0.270	0.521	0.384	0.573	0.027* (*r* = −0.67)	0.732
	M. triceps	0.996	0.146	0.668	0.633	0.559	0.059	0.432	0.053	0.748	0.620	0.980
	Shoulder elevators	0.438	0.036* (*r* = − 0.63)	0.480	0.198	0.537	0.044* (*r* = − 0.62)	0.545	0.581	0.292	0.742	0.116
	M. quadriceps	0.844	0.034* (*r* = 0.64)	0.716	0.076	0.368	0.111	0.481	0.061	0.962	0.808	0.352
	Ischiocrural muscle group	0.028 (*r* = −0.656)	0.0135	0.025* (*r* = − 0.67)	0.808	0.179	0.024 * (*r* = − 0.62)	0.863	0.002*(*r* = − 0.83)	0.701	0.00** (*r* = − 0.88)	0.663
	Upper extremity	0.302	0.132	0.181	0.635	0.741	0.047* (*r* = 0.61)	0.833	0.048* (*r* = 0.61)	0.912	0.095	0.916
	Torso	0.438	0.036* (*r* = − 0.63)	0.480	0.198	0.537	0.044* (*r* = − 0.62)	0.545	0.581	0.292	0.742	0.116
	Lower extremity	0.547	0.029* (*r* = − 0.65)	0.251	0.265		0.018* (*r* = − 0.69)	0.572	0.001** (*r* = 0.83)	0.885	0.183	0.789

^*^: *p* < 0,05

^**^: *p* < 0,001

*Vtotal* total injuries, *Vacute* acute injuries, *Vchron* chronic damages/complaints, *Vuex* Injuries/damages/complaints at the upper extremity, *Vlex* Injuries/damages/complaints at the lower extremity, *Vtor* Injuries/damages/complaints at the torso

For the JL, positive correlations between force and injury were calculated: With the male dancers of the JL, the higher right-sided strength in the shoulder elevators correlated with the higher total number of injuries on the right side (*p* = 0.033, *r* = 0.592) and also with a higher number of injuries of the lower extremities on the right side of the body (*p* = 0.011, *r* = 0.677).

Similarly, the higher strength of the right ischio-crural musculature positively correlated with the higher number of acute injuries on the right side of the body (*p* = 0.043, *r* = 0.567). The only negative correlation of the JL with the male dancers was between the higher maximum strength of the M. biceps on the right and the increased number of injuries on the left side of the body. No significant correlations could be calculated for the female dancers of the JL in this respect. On the other hand, comparing the JL to the EPL, there was predominantly an increased susceptibility to injury on the ipsi lateral - actually stronger – side, possibly resulting from inappropriate - or over- loading.

## Discussion

The Latin American formation dance sport has enjoyed great popularity for years. This sport discipline is associated with considerable psycho-physical - including asymmetric – loads on both sides of the body [1, 9, 16, 24, 25]. The influence of this movement characteristic on motor skills - as well as the highest risk of injury within the dance sport disciplines - have not been assessed to date. The aim of this study was therefore to analyze the side-related strength and balance capability subject to injuries, gender and performance standards. The results are supposed to better understand the effects of the specific movement elements and to reduce the injury risk.

### Isometric maximum strength and balance capability

In formation dance, the upper extremity is loaded via the right side of the body during guidance or contact, as well as the execution of the specific maximum difficulties. In this study, a significant strength difference was determined in favor of the right side of the body, especially at the upper extremity. This scientifically confirms the assumption of a functional and morphological adaptation in terms of laterality. This would coincide with the results in other dance styles - albeit the professional dance [10–12]. However, the influence of right-handedness (82%) is neglected here, which may possibly reduce the significance [26, 27]. This influence should be taken into account in future investigations.

The lower extremities are less (*M. quadriceps*) or not at all (hamstrings) affected by the rather accidental differences, which again would suggest a more even load on the lower extremities -, compared to the upper extremities - and which is supported by the results of the balance measurement. Simultaneously, it should be considered that there is a methodological limitation, influence of which on the results remains unclear.

### Injuries

Although there were only minor differences as to number of performance groups, there were even more significant differences in the further differentiation (chronic-acute, JL-EPL and m - f). In contrast to the EPL, no evidence of laterality within the JL was accounted for. The right side of the body was more frequently affected in the EPL, which can be interpreted as a consequence of the higher asymmetric load in this group. The fact that an injury risk rises with the performance level, as already described in dance medicine literature e. g. [28–30], could be confirmed with this study.

In order to cause chronic complaints, the loads must affect the body over a certain period of time. This may serve as an explanation why the dancers of the EPL suffer from chronic complaints more often than those of the JL. This has already been described by other authors investigating the dance sport [23]. The same applies to the localizations. Various studies have shown that, not only in Latin American dance, especially the lower extremities show an increased susceptibility to injury [24, 31–36]. In addition, the extreme difficulties in Latin American formation dance have to be added that comprise an increased risk of injury to shoulder, spine, pelvis and hip as demonstrated in this study [6, 17, 29].

A high susceptibility to injury of the hand and wrist was found, too. This may also be explained by the excessive guiding of the male partner and contact of the dance couple via hand. While fatigue processes favor injuries in dancing [37], it is still unclear to date - at least in formation dance - to what extent other aspects, such as anthropometric aspects (e. g. also the size/weight ratio between male and female dancers are involved [6, 38]. These could be potential issues to be addressed in future, because the dance couples of a formation have been assembled more according to aesthetic and less according to functional-preventive aspects, which can result in an increased risk of health damage if the matching of the couples is not optimal. The lack of coordination within the couple is also a major cause of injury in single couples [7].

### Balance - isometric maximum strength - injuries/chronic complaints

There was no evidence of any correlation between the balance and injuries in the EPL. In the JL, however, injuries were localized on the less well-balanced side. The technical overload in the JL could serve as an explanation due to an overload and fatigue processes of the heavily loaded right side in the young dancers [37]. In the EPL, injuries were significantly more frequent at the muscularly weaker side, which could in turn indicate a lack of compensatory capacity, e. g. in the form of a balancing training. This could be a good argument for an accompanying strength training of the upper extremity not yet included in the standard training program. The strength training would have the function of preparing for maximum difficulty in the JL and the function of compensating in the EPL. However, particular care must be taken to ensure that the aesthetic ideal image of both sexes is not endangered. In addition, further studies with larger groups of test subjects are necessary in order to support the results described in this pilot project.

### Limitations

The test subjects were recruited from one dance club which guaranteed a certain degree of homogenity. On the one hand, a cautious generalization of the results can be justified by the fact that the clubs in a national league system take part in competitions several times a year. On the other hand, an excessive differentiation of groups can have an adverse effect as calculations on a random basis may contain inaccuracies or result in fictitious earnings.

The force measurement with the power chair “m3 Diagnosis professional” offers many advantages. Due to the fixed construction of the device, the degrees of freedom during the movement are limited to a minimum and thus ensure an easy handling with a low risk of injury. Repeated runs can therefore provide a higher guarantee to achieve the same results. In the isometric measurement of torque, disturbing variables, such as the change in muscle length and the individual speed of joint movements, are largely eliminated by immobile resistance. The superiority of a better lever length can be prevented by the torque and allows to compare subjects of any body size. However, force measurements should be interpreted with caution, as the required maximum force values can be influenced by factors such as compliance, fatigue and the subject’s motivation. The influence of the strength-enhancing auxiliary musculature could be reduced by a strictly given and continuously controlled measuring position and thus a quite exact estimation of the desired musculature could be achieved.

A questionnaire with self-assessment covering a period of 24 months is critically evaluated in science [39]. In addition, dancers tend to minimize physical discomfort. Therefore, it cannot be ruled out that the results may be influenced by lacking injury data [31].

Another weak point is the small group which limits the informative value. Larger subject groups seem to increase the informative value and are suggested for future research.

## Conclusion

The partly asymmetrical, one-sided and gender-specific movement elements of the Latin American formation dance partly lead to morphological and functional asymmetric adaptations depending on the performance standard and gender. These adjustments are closely related to acute injuries and chronic complaints. This must be taken into account with the derived preventive measures. In addition to the implementation of prevention measures, further studies in the field of dance sports with larger numbers of cases and styles are required.
